# Editorial Note: Industrial structure, high-quality development of logistics industry and the economy

**DOI:** 10.1371/journal.pone.0336447

**Published:** 2025-11-11

**Authors:** The PLOS One Editors

This article [1] was identified as one of a series of submissions for which we have concerns that the peer review did not meet the expected standards for *PLOS One*. We regret that the issues were not identified and addressed prior to the article’s publication.

This note is not intended to question the quality of the article or contributions of the authors to the review process.

Additionally, the article cited in [1] as Reference 32 was retracted after publication of [1]. The authors have provided [2] as an alternative reference to replace the original Reference 32.
